# The Impact of COVID‐19 on Dental Hygienists' Stress and Workplace Conditions in Japan

**DOI:** 10.1111/idh.70015

**Published:** 2025-12-07

**Authors:** Rieko Takemae, Hiroteru Okamoto, Masanari Takemae, Zac Morse

**Affiliations:** ^1^ Tokyo Nishinomori Dental Hygienist School Tokyo Japan; ^2^ Department of Health and Welfare, Faculty of Health Sciences Kyorin University Tokyo Japan; ^3^ College of Dentistry American University of Iraq‐Baghdad Baghdad Iraq

**Keywords:** dental hygiene, mental health, occupational stress, pandemics, personal protective equipment, working conditions

## Abstract

**Objectives:**

The COVID‐19 pandemic emerged in late 2019 and led to unprecedented global lockdowns, disrupting numerous industries, including dentistry. This study aimed to investigate the practicing conditions, personal protective equipment (PPE) usage, and impact of the pandemic on dental hygienists (DHs) in Japan, where strict lockdowns were not enforced but compliance to reduce infection spread was high, and healthcare clinics remained open.

**Methods:**

This cross‐sectional study utilised an online survey distributed to 354 dental hygiene vocational school graduates in Tokyo, Japan, between May 9 and June 5, 2022. The questionnaire, adapted from a New Zealand Dental & Oral Health Therapists Association survey, collected data on demographics, practicing conditions, the impact of COVID‐19 on DHs, personal protective equipment usage, and stress levels during different COVID‐19 waves.

**Results:**

Out of 354 DHs, 77 (21.8%) provided valid responses. Most (94.8%) worked in Tokyo and neighbouring areas, with 85.7% working at private institutions. The COVID‐19 pandemic led to ceasing work (11.3%), reduced working days (27.3%), decreased patient visits (49.4%), and changes in personal protective equipment usage. High‐stress levels peaked during the first wave (46.8%) and fell during the fifth (37.7%) and sixth (11.7%) waves. The most common stress factors were related to inhibiting stress relief, infection risk, and protection.

**Conclusion:**

The COVID‐19 pandemic impacted DHs’ working conditions, personal protective equipment usage, and stress levels in Japan. Despite not facing a strict lockdown, DHs, who have experienced detrimental changes in the work environment, will need post‐pandemic support that considers droplet infection protection and practitioner well‐being.

## 1 Introduction

The novel coronavirus disease (COVID‐19) emerged in Wuhan City, China, in December 2019 and was declared a Public Health Emergency of International Concern (PHEIC) by the World Health Organization (WHO) on January 30, 2020 [1]. As the virus spread globally with significant consequences, the WHO declared a global pandemic on March 11, 2020 [2]. Following this announcement and the unprecedented health crisis, numerous regions implemented lockdowns to curb the virus's transmission. By the end of March 2020, over 100 countries had enacted full or partial lockdowns, affecting billions of people worldwide. These measures disrupted various aspects of society, such as education, the economy, trade, employment, and health [3].

The COVID‐19 pandemic has had a significant impact on healthcare workers around the world [4]. The field of dentistry was also severely impacted by the pandemic, with reports of clinic closures, physical and mental stress among dental care workers, and challenges in infection prevention [5, 6, 7, 8, 9, 10, 11, 12]. By December 2022, Japan had experienced eight waves of COVID‐19 infections (Appendix S1) [13]. As a result, the Tokyo Metropolitan Government declared a state of emergency four times and implemented priority measures to prevent the spread of the virus on two occasions by the end of the sixth wave in June 2022 (Table 1) [14]. However, unlike the lockdowns imposed in other countries, the Japanese government's state of emergency declarations relied on voluntary cooperation with public health measures, rather than strict compliance, as these declarations did not provide legal authority to enforce strict lockdowns or issue fines for noncompliance [15, 16]. Nevertheless, social pressure and the collective desire to control the virus's spread often resulted in high compliance despite the lack of legal enforcement [17].

**TABLE 1:** Timeline and characteristics of COVID‐19 waves in Tokyo, Japan: state of emergency declarations, priority measures, and predominant variants.

COVID‐19 wave number in Japan	Wave periods	Declarations for state of emergency in Tokyo	Priority measures to prevent infection spread in Tokyo	COVID‐19 variant strain
First wave	13th to 20th week in 2020	First—April 7 to May 25 2020		
Second wave	26th to 39th week in 2020			
Third wave	44th week in 2020 to 8th week in 2021			
Fourth wave	9th to 24th week in 2021	Second—January 8 to March 21 2021, Third—April 25 to June 20 2021	First—April 12 to 242021	Alpha
Fifth wave	28th to 38th week in 2021	Fourth—July 12 to September 30 2021	Second—June 21 to July 11 2021	Delta
Sixth wave	51st week in 2021 to 24th week in 2022		Third—January 21 to March 21 2022	Omicron (BA.1/BA.2)

Healthcare clinics in Japan did not close, so under these unprecedented pandemic circumstances, dental care workers' suspected physical and mental effects and their practicing conditions are relatively unknown. Throughout the pandemic, healthcare clinics in Japan remained open, leaving the potential physical and mental impact on dental care professionals and their working conditions relatively unexplored [18]. This research aimed to investigate the practice conditions, personal protective equipment (PPE) usage, and other impacts on dental hygienists (DHs) before and after the onset of the COVID‐19 pandemic in Japan.

## 2 Study Population and Methodology

This study employed a cross‐sectional design using an anonymous online survey. Ethics approval for the study was granted by the Research Ethics Review Committee of the Japanese Dental Hygiene Education Society (approval number: sdheR3‐03). A convenience sample of dental hygiene vocational school graduates in Tokyo, Japan, was recruited. The alumni association possessed email addresses of dental hygienists who had graduated in 2013 or later. Only graduates with available email contacts were invited to participate in the survey. One week before the survey launch, a study information email was sent to these graduates, detailing the study's purpose, ethics information, and how the survey would be conducted. Recipients were informed that they could opt out of further communication regarding the study by replying to the email; however, no opt‐out requests were received. The online survey was conducted over a 4‐week period, from May 9 to June 5, 2022. A total of 354 DHs (all female) were invited to participate. Participants received a link to the questionnaire hosted on Microsoft Forms, along with an explanation of the study and a request for participation. Informed consent was obtained electronically through the voluntary completion of the survey and by answering the first question, which specifically asked whether the participant wished to participate in the study. No incentives to participate were offered. The survey was designed to prevent the collection of cookies, IP addresses, and log histories to ensure participant privacy and data protection. The principal investigator is responsible for data management, and survey data stored on a USB drive will be kept securely locked for 5 years post‐study. After this period, the data will be erased using specialised software, and the USB drive will be physically destroyed.

### 2.1 The Questionnaire

As no established instruments were available, the survey was based on a New Zealand Dental & Oral Health Therapists Association COVID‐19 survey conducted in 2020 and 2021 (unpublished data). With permission, three Japanese investigators translated the questionnaire into Japanese and adapted it to the Japanese context. The survey comprised 26 questions on one page hosted on Microsoft Forms, including open‐ and closed‐ended questions. The questions addressed participants' demographics, practice conditions, the impact of the COVID‐19 pandemic on dental hygienists, and the use of personal protective equipment before and after the pandemic. Additionally, the survey inquired about stress levels and major stressors during the first, fifth, and sixth COVID‐19 waves. The Visual Analogue Scale (VAS) has been widely used to assess perceived stress in workers [19]. The low‐stress group was defined as those reporting a VAS score of 0–3 points, moderate‐stress group 4–7 points, and high‐stress group 8–10 points. Before administering the electronic questionnaire, four dental hygiene teachers tested its usability and technical functionality.

### 2.2 Data Analysis

Data were compiled and analysed using IBM SPSS Statistics software (Version 26.0 for Windows). Differences among categorical variables were assessed using the chi‐square test. For continuous dependent variables, a one‐way analysis of variance (ANOVA) was conducted. Upon identifying significant differences with ANOVA, post hoc analyses were executed using the Bonferroni correction. All statistical conclusions were based on a significance threshold of *p* < 0.05.

## 3 Results

Out of the 354 dental hygienists who received the survey, 100 responded, yielding a response rate of 28.2% (100 ÷ 354). However, only 77 of the 100 responses were valid, resulting in an effective response rate of 21.8% (77 ÷ 354). The remaining 23 responses were excluded from the analysis due to incomplete data (*n* = 21) and lack of consent (*n* = 2). The following results are based on the 77 valid responses.

Table 2 displays the demographics and practice conditions of the respondents. Most respondents worked in Tokyo and the surrounding areas, including Saitama, Kanagawa, and Chiba, and most were employed at private institutions. Among the respondents, nine out of 77 dental hygienists (11.3%) had ceased working, and eight (10.4%) experienced lower pay than before the COVID‐19 pandemic. In addition, relative to pre‐pandemic conditions, 21 dental hygienists (27.3%) reported a decrease in workdays, and 38 (49.4%) noted a decline of 10 or more patients per week (27.3%).

**TABLE 2:** Work conditions since the COVID‐19 pandemic of dental hygienists in Japan (*n* = 77).

	*n*	%
Workplace		
Tokyo, Saitama, Kanagawa, Chiba	73	94.8
Others	4	5.2
Work facility		
Private dental institutions	66	85.7
Public dental institutions	2	2.6
Community‐based dental care	9	11.7
Closed dental care since the first COVID‐19 pandemic		
Have closed the dental practice	9	11.7
Have not closed the dental practice	68	88.3
Salary since the first COVID‐19 pandemic		
Have had pay cut, late payment or not been paid	8	10.4
Paid the same before the pandemic	69	89.6
Attendance status since the first COVID‐19 pandemic		
Have worked less days than before the pandemic	21	27.3
Worked the same days as before the pandemic	56	72.7
Number of patients since the first COVID‐19 pandemic		
Number of patients decreased by < 10 per week than before the pandemic	38	49.4
Same number of patients as before the pandemic	36	46.8
Number of patients increased by > 10 per week before the pandemic	3	3.9

Tables 3 and 4 present the usage of personal protective equipment (PPE). There was no change in mask and glove usage during the pandemic, but 44 (57.1%) dental hygienists double‐masked or 26 (33.8%) double‐gloved. Rates of wearing face shields, gowns, caps, and N95 masks during the pandemic increased substantially compared to before the pandemic (Table 3). Prior to the COVID‐19 pandemic, 26 (33.8%) dental hygienists wore masks and gloves, while 23 (29.9%) wore masks, gloves, and goggles (Table 4). Among the 26 (33.8%) dental hygienists who wore masks and gloves before the pandemic, the following PPE changes were made due to COVID‐19: 25 adopted face shields, 17 double‐masked, 10 double‐gloved, and 10 added gowns (Table 4). Of the 23 dental hygienists who wore masks, gloves, and goggles pre‐pandemic, 18 adopted face shields, 17 double‐masked, 10 double‐gloved, and 10 added gowns due to COVID‐19 (Table 4).

**TABLE 3:** PPE worn routinely by dental hygienists in Japan before and during the COVID‐19 pandemic (multiple answers) (*n* = 77).

PPE	Before pandemic		During pandemic		Chi‐squared test
	*n*	%	*n*	%	
Mask	70	90.9	70	90.9	ns
Mask of two layers	—	—	44	57.1	—
Gloves	70	90.9	70	90.9	ns
Gloves of two layers	—	—	26	33.8	—
Goggles	35	45.5	28	36.4	ns
Face shield	21	27.3	55	71.4	*p* < 0.01
Gown	3	3.9	21	27.3	*p* < 0.01
Cap	2	2.6	17	22.1	*p* < 0.01
N95 mask	1	1.3	11	14.3	*p* < 0.01
No PPE	1	1.3	0	0.0	—
No PPE added	0	0.0	12	15.6	—

Abbreviations: ns, not significant; PPE, personal protective equipment.

**TABLE 4:** Combination of PPE worn routinely by dental hygienists in Japan before the COVID‐19 pandemic and additional PPE during the pandemic (*n* = 77).

PPE combinations	Before pandemic		Additional PPE during the pandemic						
			Mask (including wearing two layers)	Gloves (including wearing two layers)	Goggles	Face shield	Gown	Cap	N95 mask
	*n*	%	*n*	*n*	*n*	*n*	*n*	*n*	*n*
Mask + gloves	26	33.8	17	10	8	25	10	4	4
Mask + gloves + goggles	23	29.9	17	10	—	18	10	10	4
Mask + gloves + face shield	7	9.1	6	3	3	—	1	1	1
Mask + gloves + goggles + face shield	5	6.5	2	2	—	—	—	1	0
Mask + gloves + goggles + face shield + cap	1	1.3	0	0	—	—	0	—	0
Mask + gloves + goggles + gown + N95 mask	1	1.3	0	0	—	—	—	0	—
Mask + gloves + face shield + gown + cap	1	1.3	0	0	—	—	—	—	0
Mask + gloves + face shield + gown	1	1.3	0	0	—	—	—	0	0
Mask + face shield	2	2.6	0	0	0	—	0	0	0
Mask + goggles	2	2.6	1	0	—	1	0	0	1
Gloves + goggles + face shield	2	2.6	0	0	—	—	0	0	0
Gloves + face shield	1	1.3	0	0	0	—	0	0	0
Gloves + goggles	1	1.3	0	0	—	1	0	0	1
Others	4	5.2	1	1	2	2	0	1	0

Abbreviation: PPE, personal protective equipment.

During the first wave of the COVID‐19 pandemic, 36 (46.8%) dental hygienists had high‐stress mean levels (i.e., stress scores of 8–10), and 35 (45.5%) had moderate‐stress levels (i.e., stress scores of 4–7), which were significantly higher (*p* < 0.01 using Bonferroni correction) than the six (7.8%) with low‐stress levels (i.e., stress scores of 0–3) (Table 5). In the fifth and sixth waves, mean high‐ and middle‐level stress groups were significantly higher than the low‐stress level group (*p* < 0.01 using Bonferroni correction) (Table 5).

**TABLE 5:** Stress levels among Japanese dental hygienists during different COVID‐19 waves (*n* = 77).

Wave of the COVID‐19 epidemic	Stress group	*N*	Mean	SD	ANOVA	Multiple comparisons using the Bonferroni method
The first wave of the COVID‐19 epidemic	Low stress (0–3)	6	1.83	1.17	*p* < 0.01	Low vs. middle groups *p* < 0.01
	Middle stress (4–7)	35	5.83	1.07		Middle vs. high groups *p* < 0.01
	High stress (8–10)	36	9.17	0.91		Low vs. high groups *p* < 0.01
The fifth wave of the COVID‐19 epidemic	Low stress (0–3)	7	1.86	1.35	*p* < 0.01	Low vs. middle groups *p* < 0.01
	Middle stress (4–7)	41	5.57	1.07		Middle vs. high groups *p* < 0.01
	High stress (8–10)	29	9.10	0.94		Low vs. high groups *p* < 0.01
The sixth wave of the COVID‐19 epidemic	Low stress (0–3)	12	1.75	0.97	*p* < 0.01	Low vs. middle groups *p* < 0.01
	Middle stress (4–7)	56	5.64	1.02		Middle vs. high groups *p* < 0.01
	High stress (8–10)	9	8.56	0.88		Low vs. high groups *p* < 0.01

**FIGURE 1:**
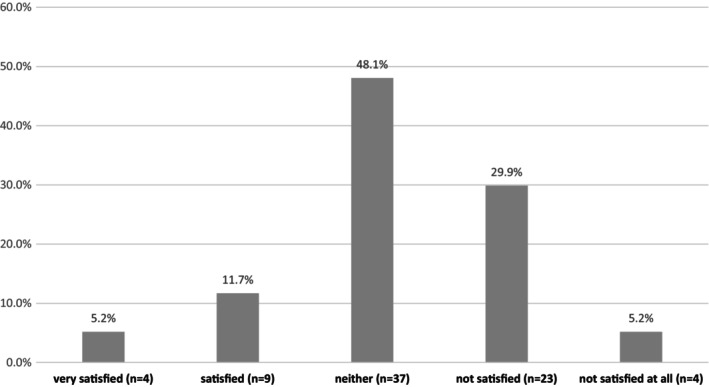
Satisfaction with workplace infection prevention measures in Japanese dental hygienists after the first COVID‐19 wave (*n* = 77).

**FIGURE 2:**
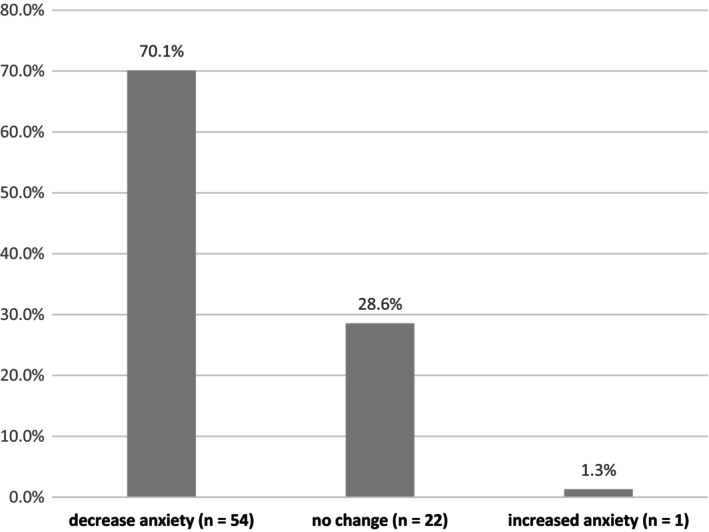
Changes in fear and worry in Japanese dental hygienists between the fifth and sixth COVID‐19 waves (*n* = 77).

Since the onset of the pandemic, 27 dental hygienists (35.1%) expressed dissatisfaction with stress management in the workplace, while 13 (16.9%) expressed satisfaction (Figure 1). At the time of the survey (during the sixth wave), 54 dental hygienists (70.1%) reported experiencing fewer worries and anxieties about COVID‐19 infections compared to the previous fifth wave (Figure 2).

The most prevalent stress factors for dental hygienists during the COVID‐19 pandemic were ‘stress factors related to inhibiting stress relief’ (26 [33.8%] respondents), ‘stress factors related to infection risk’ (16 [20.8%] respondents), ‘stress factors associated with infection protection’ (11 [14.3%] respondents) and ‘stress factors related to getting infected or infecting others’ (5 [6.5%] respondents) (Table 6).

**TABLE 6:** Major stress factors for dental hygienists in Japan during the COVID‐19 pandemic (includes multiple answers) (*n* = 77).

**Stress factors inhibiting stress relief**	***n* = 26**
Can't go/restricted from going out	12
Can't go abroad or travel/can't go out to play	10
An environment that does not allow stress to be released	4
Short business hours for restaurants	2
Can't meet my friends/can't play with my friends	6
Staying home	3
**Stress factors associated with infection risk**	***n* = 16**
Fear of infection/risk of infection	5
Scaling work/droplets from scaling	4
Inadequate infection control in the workplace	3
Lack of masks/gloves	2
Commuting with a high chance of infection	1
Fear of infection at home	1
**Stress factors associated with infection protection**	***n* = 11**
Wearing a mask/wearing mask life	6
Effort/care for infection control	2
Air conditioning is not effective because windows opened for ventilation	1
Heat from wearing personal protective equipment	1
Discomfort/frustration associated with wearing personal protective equipment	1
**Stress factors related to the impact of becoming infected**	***n* = 5**
Risk of bringing infection to visiting destinations/fear of infecting older people and high‐risk patients	4
Fear of being infected and having a negative impact on work	1

## 4 Discussion

The COVID‐19 crisis led to a decrease in working days, and fewer patients were seen by the surveyed dental hygienists. Nevertheless, about 90% of dental practices remained open, operating as close to normal as possible. Although Japan did not enforce strict lockdowns, the state of emergency and priority measures to prevent infection spread discouraged people from leaving their homes. However, they could still seek and receive healthcare. In contrast, dental hygienists in some other countries were obliged to restrict their practice due to lockdowns except for emergency treatment, and some lost or changed jobs [20]. In addition, there was dissatisfaction that the payment of COVID‐19 financial support from the government was insufficient [21], and in some countries, there was concern about the economic impact on dental hygienists as their salaries were reduced due to the decreased number of patients even after COVID‐19 restrictions were eased [22, 23]. In Japan, even though there was a decrease in the number of patients consulted, as patients likely avoided visiting the clinic for fear of infection, only a small proportion of dental hygienists (approximately 10% of respondents) experienced salary reductions during the pandemic, and a decrease in income was not identified as a major stress factor.

An online survey conducted by the Ministry of Health, Labor and Welfare in September 2020 found that most respondents felt anxious and stressed about their own and their family's infection [24]. Dental hygienists, exposed to a work environment with potential aerosol and droplet infections, received education in infection prevention and PPE usage. As a result, their anxiety and stress about infection were likely lower than in the general public. In addition, in this web survey, infection prevention actions such as washing hands, wearing a mask, keeping a distance from others, and staying at home as much as possible accounted for the highest number of answers about how to relieve anxiety and stress [24].

Before the pandemic, Japanese dental hygienists primarily used PPE consisting of masks and gloves or masks, gloves, and goggles. During the pandemic, they added face shields, double masks, double gloves, and gowns to their PPE regimen. Despite these changes, 27 dental hygienists (35.1%) were dissatisfied with their response to stress in the workplace. Infection risk was identified as the second stress factor for dental hygienists, with the lack of PPE availability potentially contributing to stress.

During the COVID‐19 pandemic in Japan, the Employee Cohort Study revealed that healthcare workers' mental health was particularly poor compared to other workers in the ‘first wave’ of COVID‐19 infections [25]. High‐stress levels among dental hygienists peaked during the first wave of COVID‐19 and decreased in subsequent waves. However, mid‐level stress increased during the fifth and sixth waves. As people adapted to daily life with infection prevention measures, and the development and uptake of COVID‐19 vaccines in Japan led to a decrease in COVID‐19‐related deaths, stress and anxiety about the virus diminished. Additionally, during the sixth wave, when the survey was conducted, social activity restrictions were gradually eased, further reducing stress.

The primary stress factor for Japanese dental hygienists was the inability to release stress due to restrictions on going out, travelling, eating at restaurants, seeing family and friends, and participating in other leisure activities [24]. The first wave of COVID‐19 was particularly stressful, with limited information on the novel virus and a high mortality rate. Dental practices with a higher risk of droplet infection continued operating under these difficult circumstances, while social activities were restricted, leaving dental hygienists with fewer opportunities to relieve stress.

Among stress factors related to infection protection, wearing PPE was one of the frequently reported sources of stress. To counter discomfort caused by wearing PPE for extended periods, it is necessary to manage work time by providing regular breaks for putting on and taking off PPE. Taking appropriate infectious disease control measures that minimise stress is crucial for maintaining workers' mental health [26].

Stress and anxiety about becoming infected with COVID‐19 and infecting older individuals and patients were common among both the general public and healthcare workers, including nurses [24, 27]. With Japan having one of the highest ageing populations globally, with 28.6% of the population aged 65 and over, both the general public and medical staff have increased opportunities to interact with older individuals, which may contribute to elevated stress levels.

Healthcare workers were already more psychologically stressed than other workers before the COVID‐19 pandemic [28], and women have been recognised to be more susceptible to mental health issues during the COVID‐19 pandemic [29]. As 99% of dental hygienists in Japan are women, creating a supportive workplace environment and system that provides mental health care is needed.

The survey covered a wide range of topics that provide valuable insights into the impact of the COVID‐19 pandemic on dental hygienists in Japan, addressing an under‐researched area. However, study limitations may affect the generalisability of the findings.

The effective response rate of 21.8% in this study is comparable to the 37.9% online survey response rate of the Japanese National Census conducted by the Statistics Bureau of Japan [13]. Previous research reported response rates of 42.5% for mailed questionnaires and 27.4% for online questionnaires [30]. Japanese research into survey responses is limited, and in engineering research, average response rates were 22.6% when there was an incentive, compared to 17.8% without [31]. Reasons for non‐participation in our study were not obtained, limiting our understanding of potential response biases. Several factors could have influenced the response rate, including increased workload and stress during the pandemic, and the potential decline in email usage due to the rising popularity of social networking services like LINE in Japan. Graduates with available email contacts were only invited, potentially excluding those without up‐to‐date email information in the alumni database. Other limitations include the use of a non‐validated survey, potential self‐reporting biases, and possible volunteer and recall biases. Question‐order bias cannot be excluded, as questions were not randomised to maintain a logical flow for respondents.

The cross‐sectional nature of the study limits causal inferences, and the lack of pre‐pandemic data hinders an accurate assessment of the pandemic's impact. The study's focus on specific aspects of the pandemic's impact on DHs may have overlooked other relevant factors. The convenience sampling of graduates from a specific Tokyo vocational school may limit generalisability to other regions of Japan and other countries.

Despite the limitations, this study provides valuable information on the significant impact of the COVID‐19 pandemic on an under‐researched group, DHs in Japan, highlighting the need for further research and support for this essential workforce. It serves as a foundation for future studies to address knowledge gaps and explore the long‐term effects of the pandemic on dental hygienists in Japan.

## 5 Conclusion

The COVID‐19 pandemic has significantly impacted dental hygienists in Japan, revealing substantial challenges related to working conditions, PPE usage, and elevated stress levels. This highlights the need for comprehensive support systems to enhance workplace safety and mental health support. Future research should investigate the pandemic's long‐term impact on the profession, and evaluate strategies and interventions to strengthen resilience and preparedness to mitigate the impacts of similar future global health crises effectively. Collaborative efforts among dental practice owners, professionals, and healthcare organisations are crucial for prioritising mental health and well‐being, building comprehensive support for dental staff, and fostering preparedness for future pandemics.

## 6 Clinical Relevance

### 6.1 Scientific Rationale for Study

Given a high exposure risk, this study investigated the impacts of COVID‐19 on the working conditions and stress levels of dental hygienists in Japan.

### 6.2 Principal Findings

Significant impacts included increased PPE usage and elevated stress levels, despite the lack of strict lockdowns. Key stress factors were the inability to release stress, infection risk, and challenges to infection protection.

### 6.3 Practical Implications

Implementing measures to reduce the risk of droplet infection can create a safer and more comfortable working environment, potentially mitigating stress, improving well‐being, ensuring better care during health crises, and fostering resilience in healthcare systems.

## Author Contributions

R.T. made substantial contributions in conceptualisation, methodology, data analysis, interpretation, writing – original draft, critical revisions for important intellectual content, and final approval of the version to be published. H.O. contributed to conceptualisation, supervision of methodology and data analysis, critical revisions for important intellectual content, and final approval of the version to be published. M.T. contributed to conceptualisation, methodology, data analysis and interpretation, critical revisions for important intellectual content, and final approval of the version to be published. Z.M. contributed to data analysis and interpretation, critical revisions for important intellectual content, and final approval of the version to be published.

## Funding

The authors have nothing to report.

## Conflicts of Interest

The authors declare no conflicts of interest.

## Supporting information


**Appendix S1:** New confirmed daily COVID‐19 cases in Japan until December 30, 2022.

## Data Availability

The data that support the findings of this study are available from the corresponding author upon reasonable request.
