# Supplementary material for: The Impact of COVID‐19 on Dental Hygienists' Stress and Workplace Conditions in Japan
Source: Int J Dent Hyg. 2025 Dec 7;24(1):108–15. doi: 10.1111/idh.70015 (PMC12748027; doi:10.1111/idh.70015)
Supplement: Supplementary file 1 — Appendix S1: New confirmed daily COVID‐19 cases in Japan until December 30, 2022. [file IDH-24-108-s001.docx]

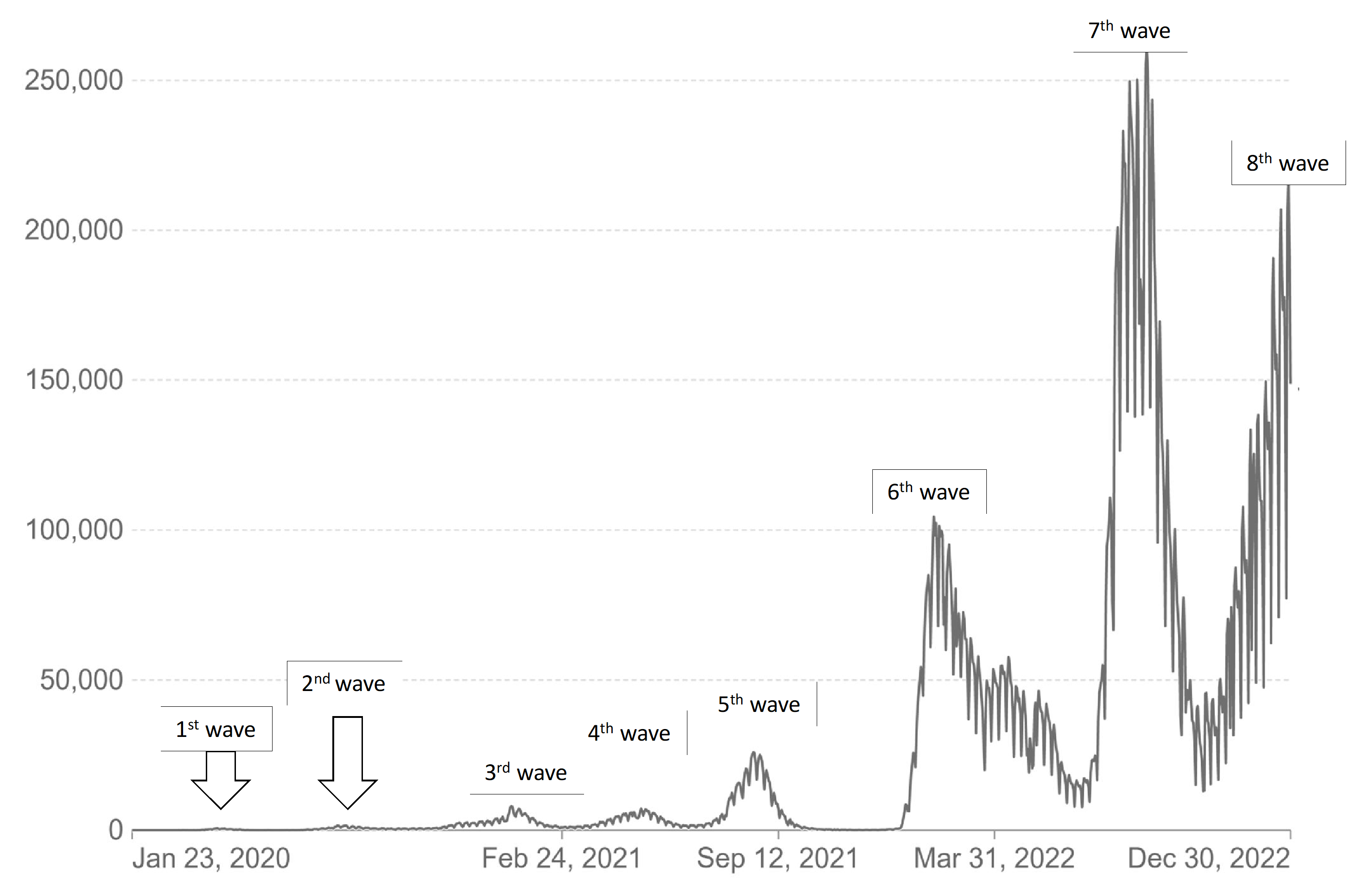


Appendix S1 New confirmed daily COVID-19 cases in Japan until December 30, 2022.

Figure was created by citing information from Our World in Data

(https://ourworldindata.org/)
